# Supplementary material for: Surface tension–driven sorting of human perilipins on lipid droplets
Source: J Cell Biol. 2024 Sep 19;223(12):e202403064. doi: 10.1083/jcb.202403064 (PMC11413419; doi:10.1083/jcb.202403064)
Supplement: Table S1 — shows antibodies used in this study. [file JCB_202403064_TableS1.docx]

**Supplementary table 1. Antibodies used in this study.**

| **Antibody** | **Reference** | **Dilution IF** | **Dilution WB** |
| --- | --- | --- | --- |
| **Anti-PLIN1 (polyclonal Rabbit)** | Abcam-ab3526 |  | 1/1000 |
| **Anti-PLIN1 (monoclonal Mouse)** | Progen-6901156S | 1/250 |  |
| **Anti-PLIN2 (polyclonal Guinea Pig)** | Progen-GP41 | 1/200 | 1/250 |
| **Anti-PLIN3 (polyclonal Rabbit)** | Sigma-HPA006427 | 1/250 |  |
| **Anti-PLIN3 (polyclonal Rabbit)** | Chromotech 10694-1-AP |  | 1/500 |
| **Anti-PLIN4 (polyclonal Rabbit)-KIAA1881** | Abcam-ab234752 | 1/250 | 1/500 |
| **Anti-HSL (polyclonal Rabbit)-D6W5S** | Cell Signaling-18381S |  | 1/500 |
| **Anti-Actin (monoclonal Mouse)** | Fisher-MA511869 |  | 1/500 |
| **Alexa anti Rabbit-488** | Fisher-A11034 | 1/500 |  |
| **Alexa anti Guinea Pig-568** | Fisher-A11075 | 1/500 |  |
| **Alexa anti mouse-647** | Fisher-A21235 | 1/500 |  |
| **Goat anti Guinea Pig, DyLight 800** | Fisher-SA5-10100 |  | 1/20000 |
| **Goat anti Mouse, DyLight 680** | Fisher-SA5-35518 |  | 1/20000 |
| **Goat anti Rabbit, DyLight 800** | Fisher-SA5-35571 |  | 1/20000 |
