# Supplementary figures and images for: Surface tension–driven sorting of human perilipins on lipid droplets
Source: J Cell Biol. 2024 Sep 19;223(12):e202403064. doi: 10.1083/jcb.202403064 (PMC11413419; doi:10.1083/jcb.202403064)

Source Data Figure 1

Fig 1A

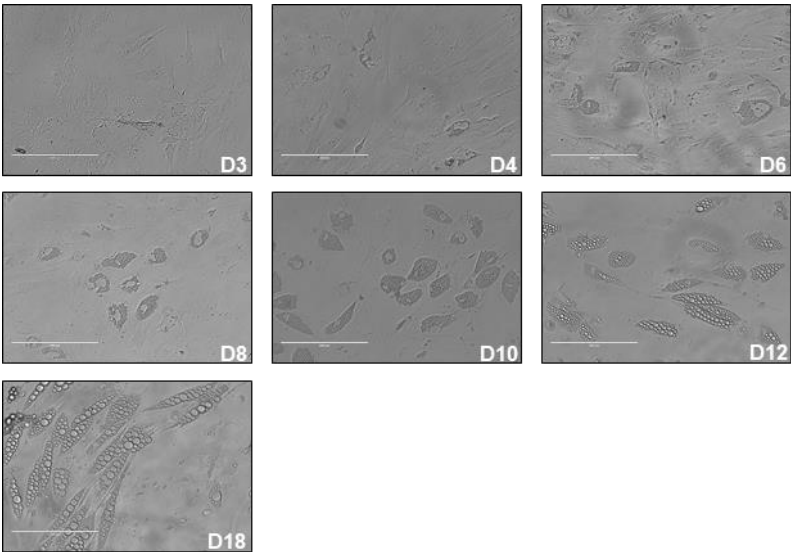

Fig 1B

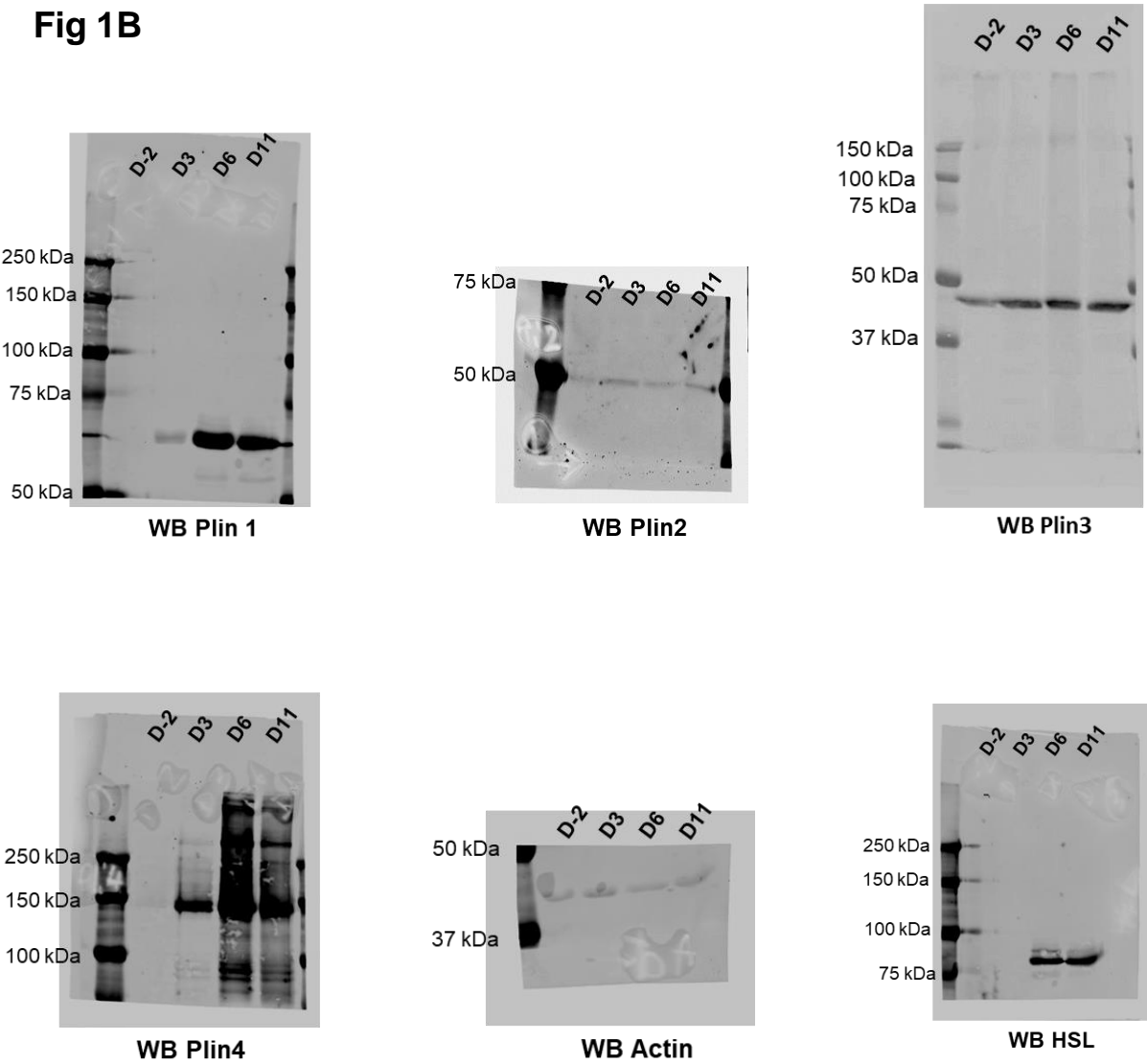

Supplement: SourceData F1 — is the source file for Fig. 1. [file JCB_202403064_SourceDataF1.pdf]

SourceDataF2B

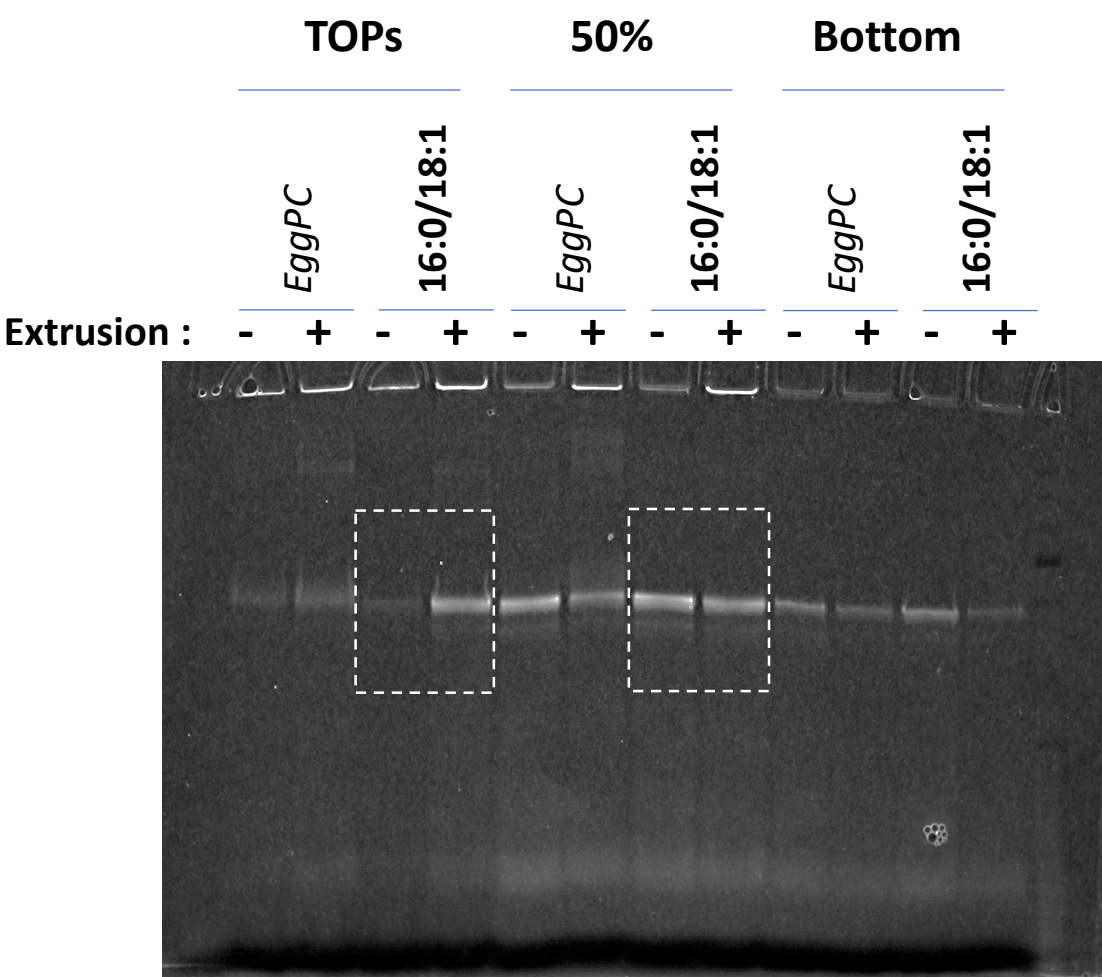

AF488 fluorescence

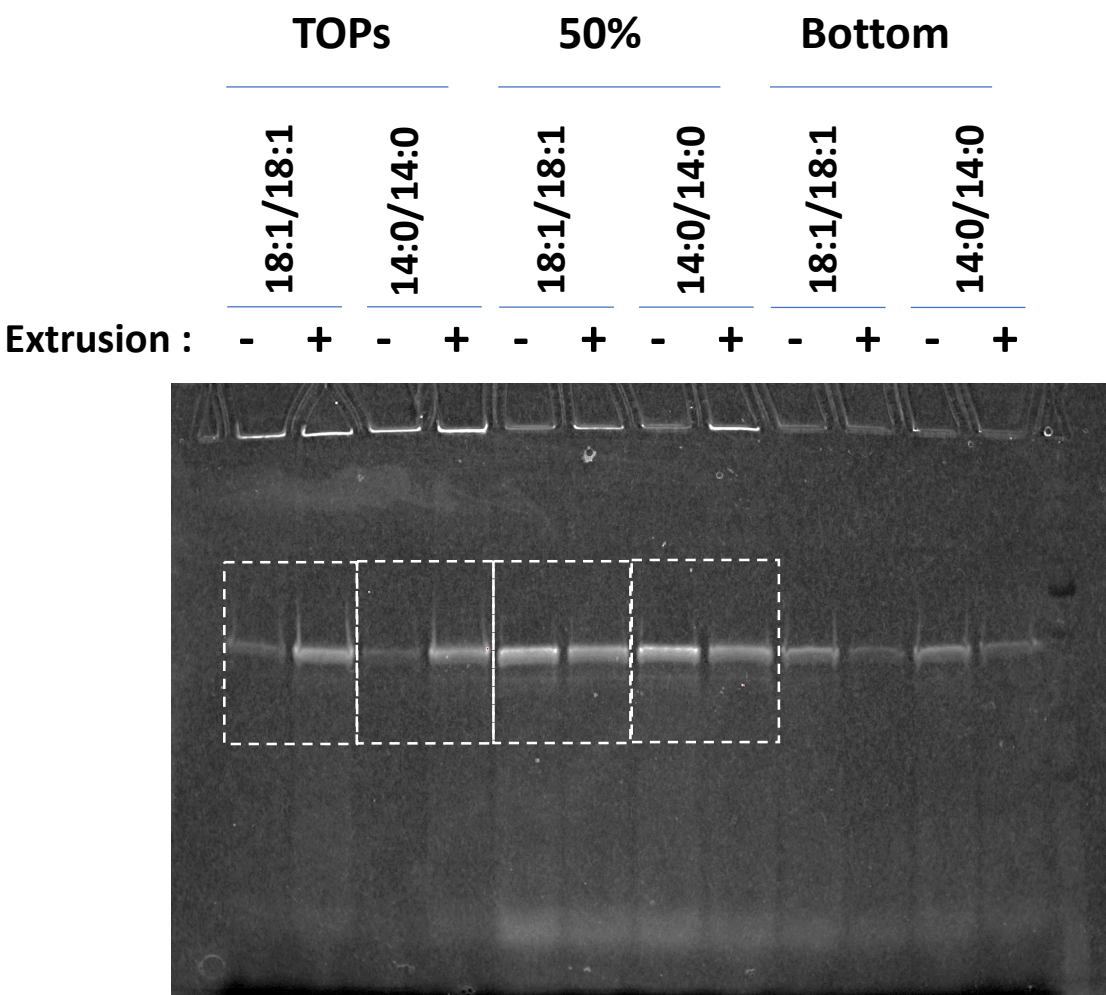

AF488 fluorescence

Supplement: SourceData F2 — is the source file for Fig. 2. [file JCB_202403064_SourceDataF2.pdf]

SourceDataF7A

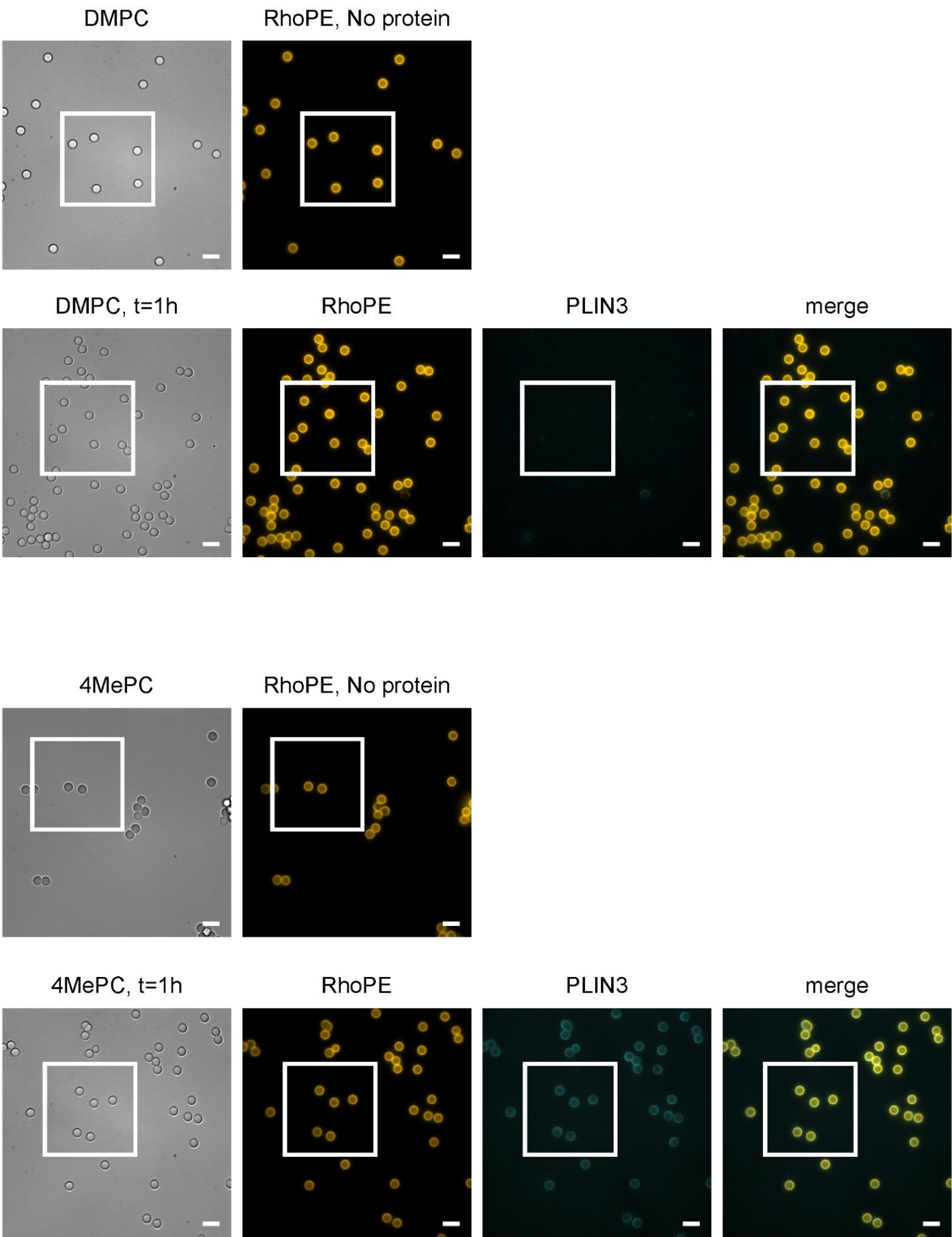

Supplement: SourceData F7 — is the source file for Fig. 7. [file JCB_202403064_SourceDataF7.pdf]

SourceDataFS2A

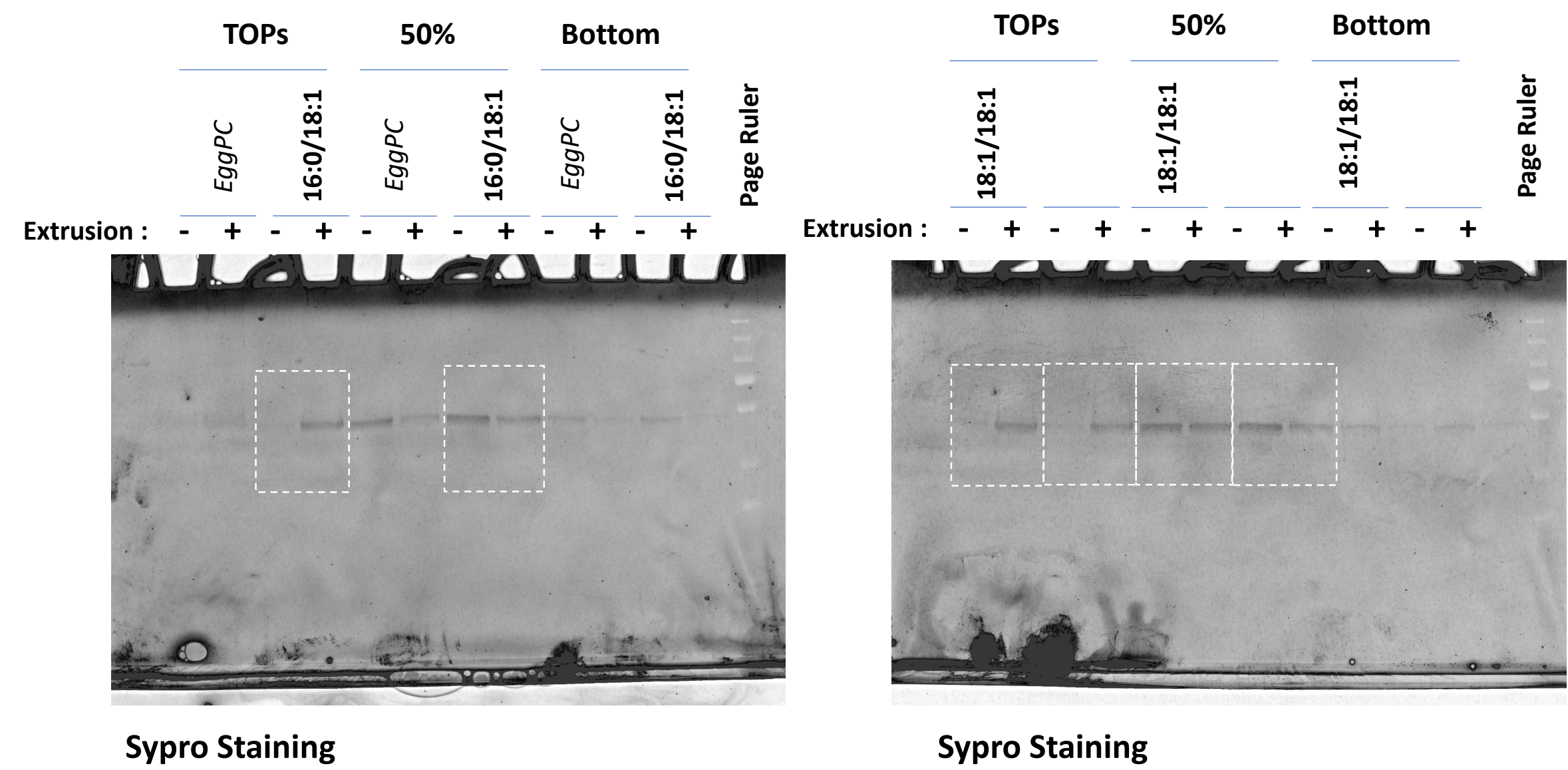

SourceDataFS2B

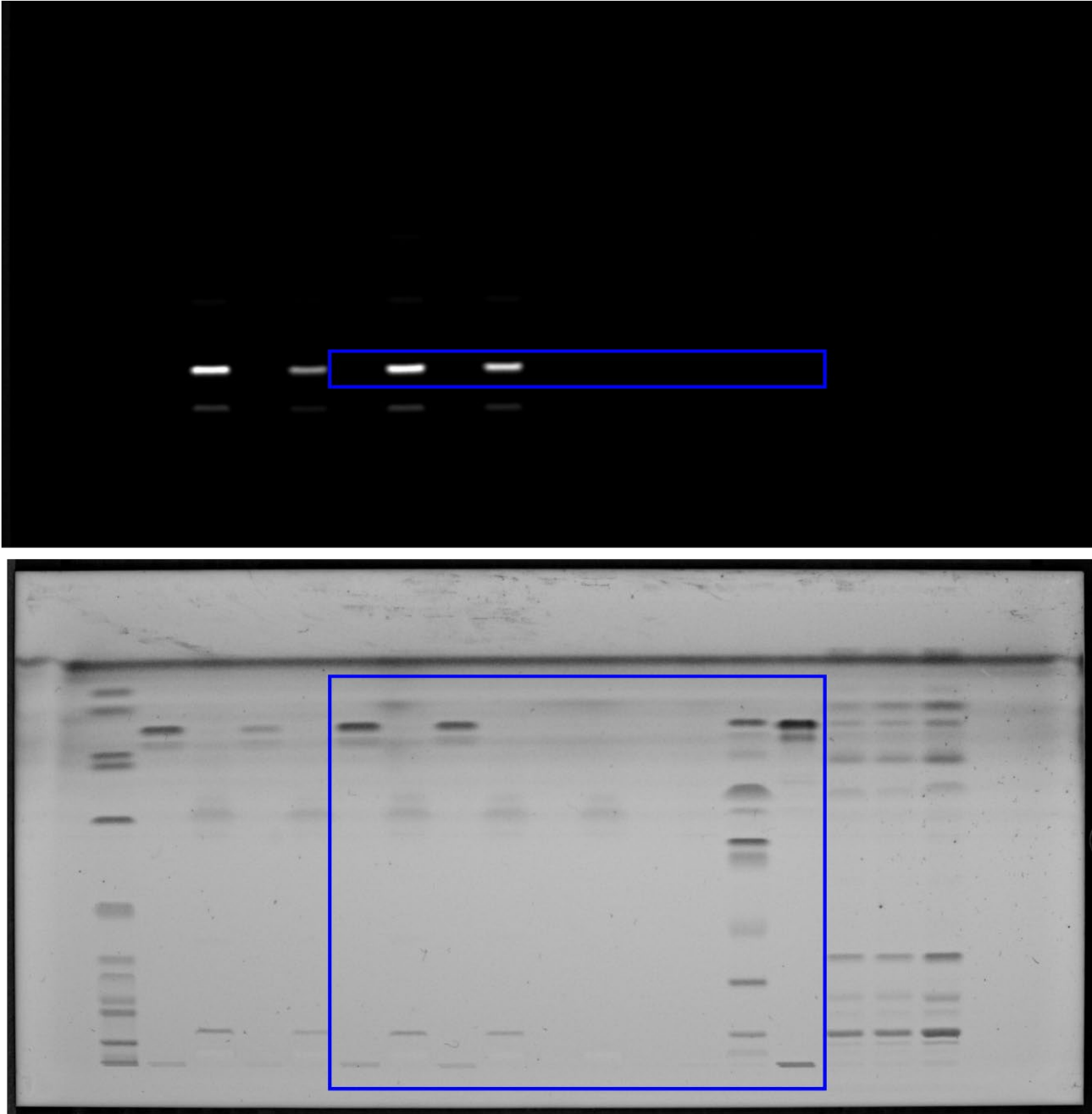

Supplement: SourceData FS2 — is the source file for Fig. S2. [file JCB_202403064_SourceDataFS2.pdf]
