# Supplementary material for: Surface tension–driven sorting of human perilipins on lipid droplets
Source: J Cell Biol. 2024 Sep 19;223(12):e202403064. doi: 10.1083/jcb.202403064 (PMC11413419; doi:10.1083/jcb.202403064)

SourceDataFS3A

GF Plin1
28.08.2023

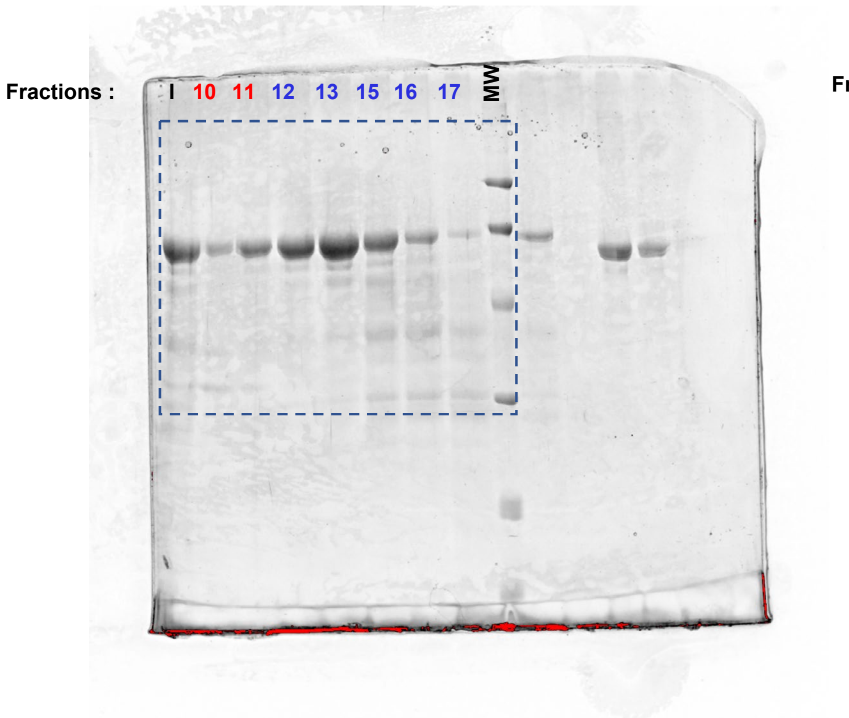

GF Plin2
27.09.2023

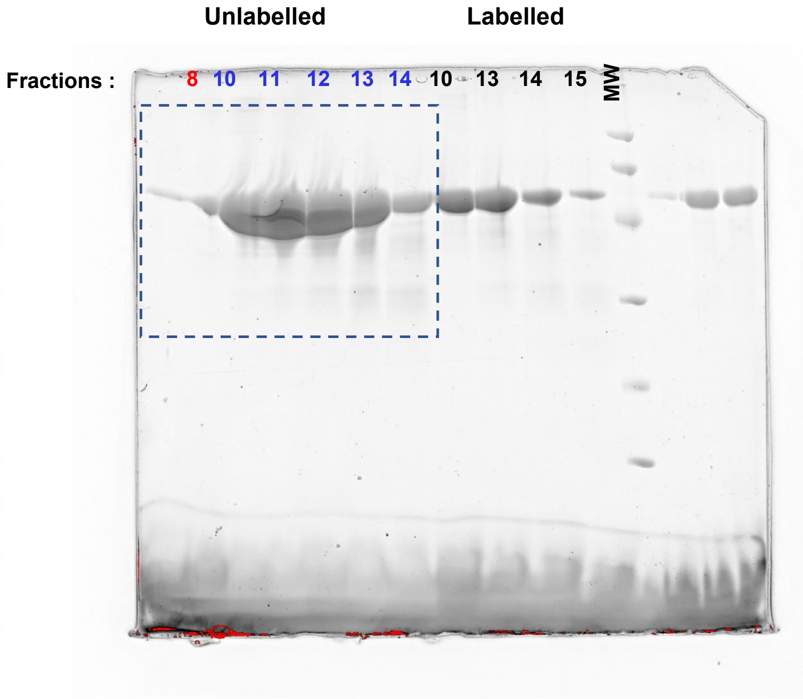

GF Plin3
03.11.2023

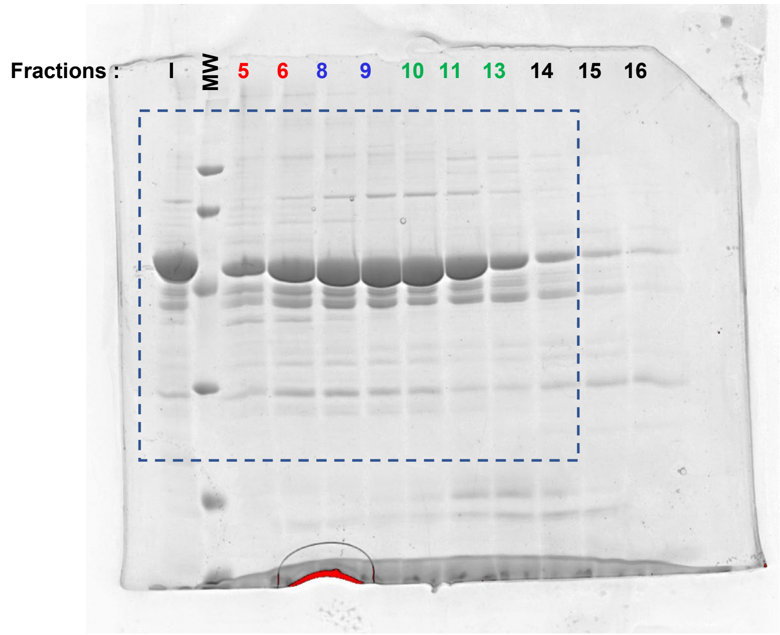

SourceDataFS3B

hPLIN1

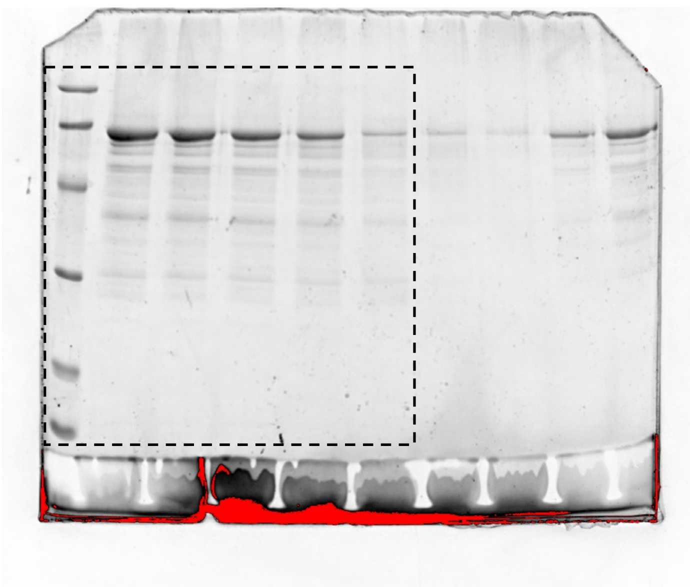

hPLIN2

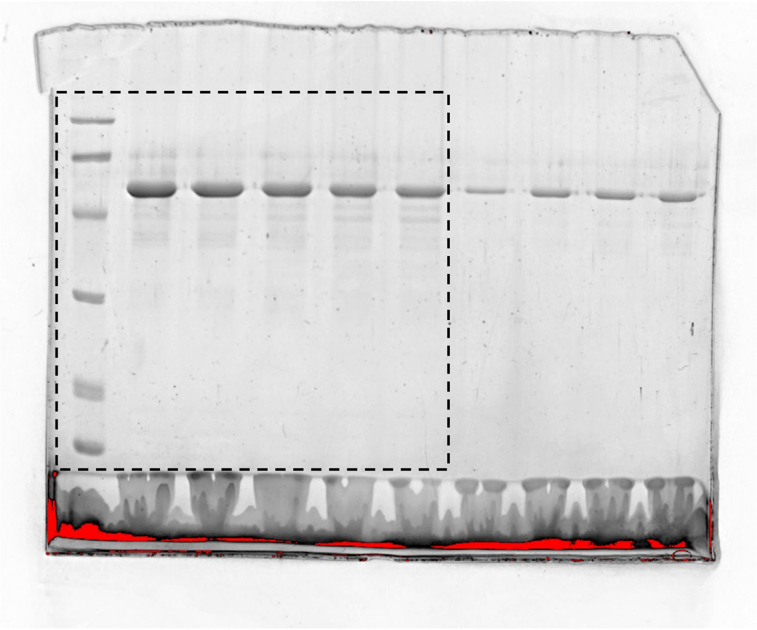

SourceDataFS3D

Limited proteolysis : 2M Urea vs no Urea

hPLIN1

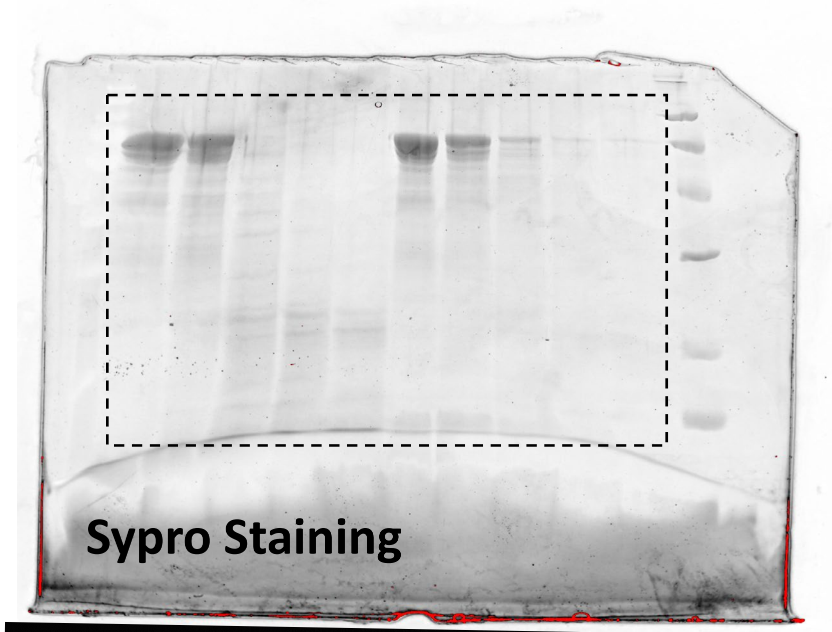

hPLIN2

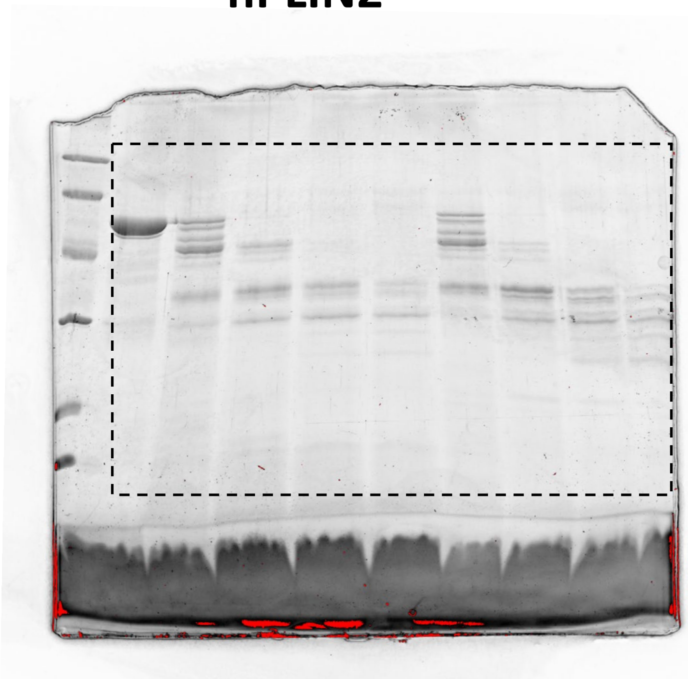

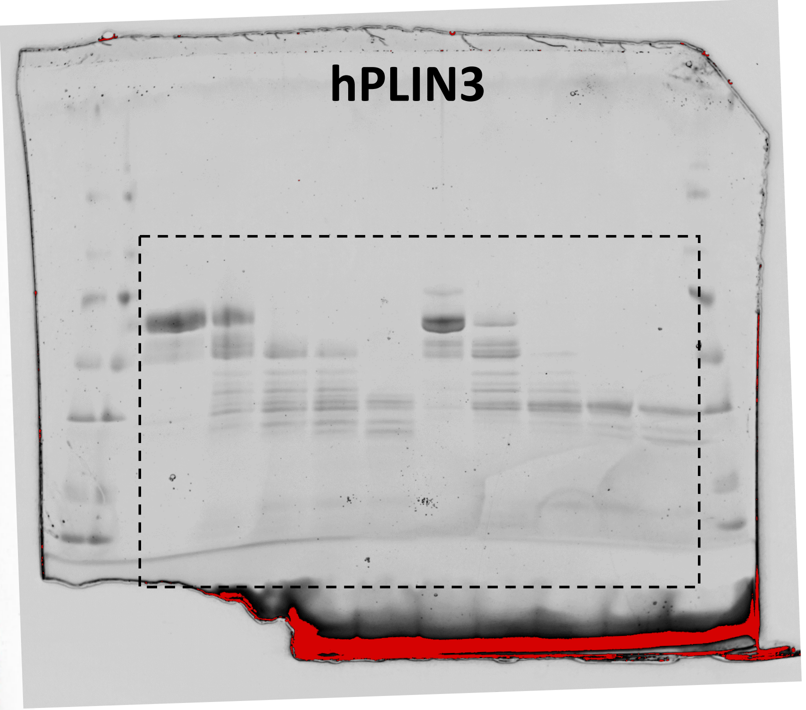

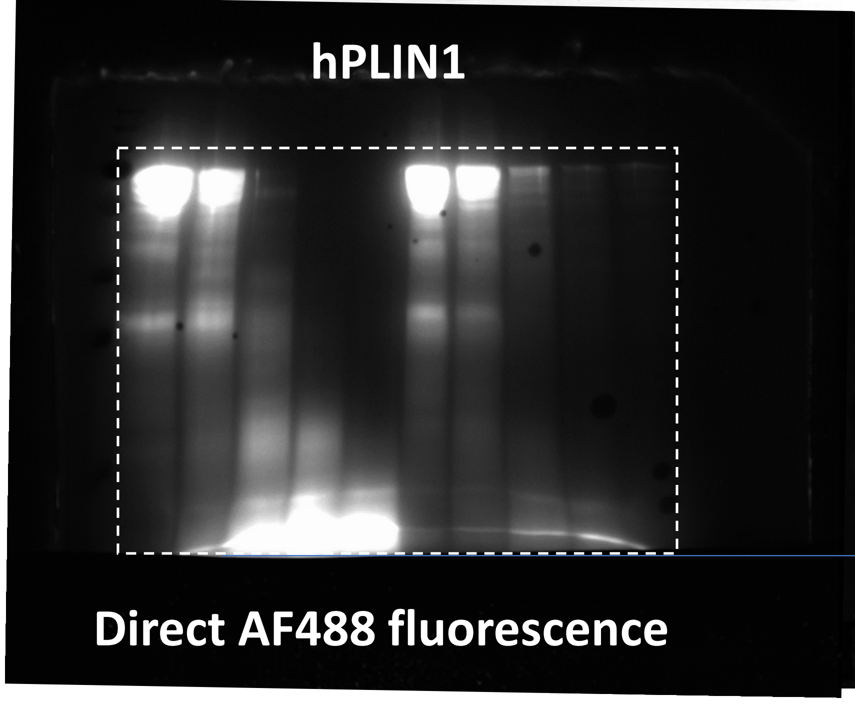

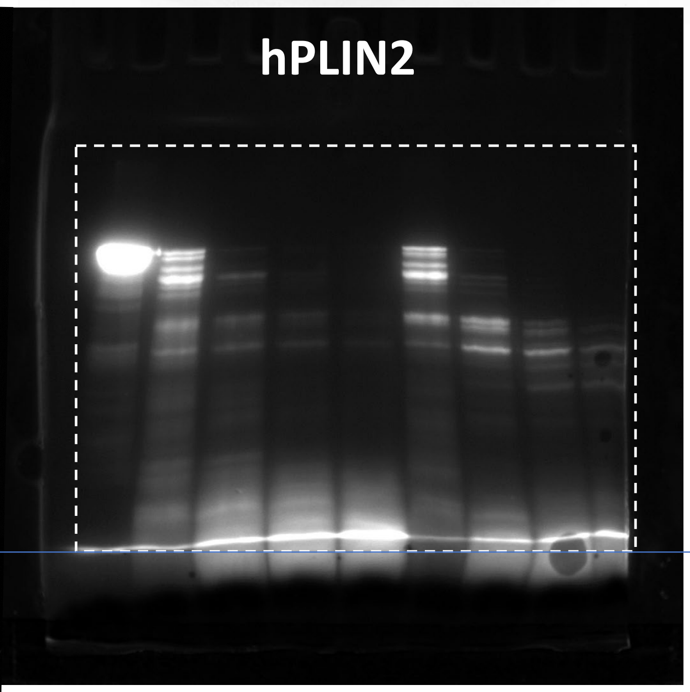

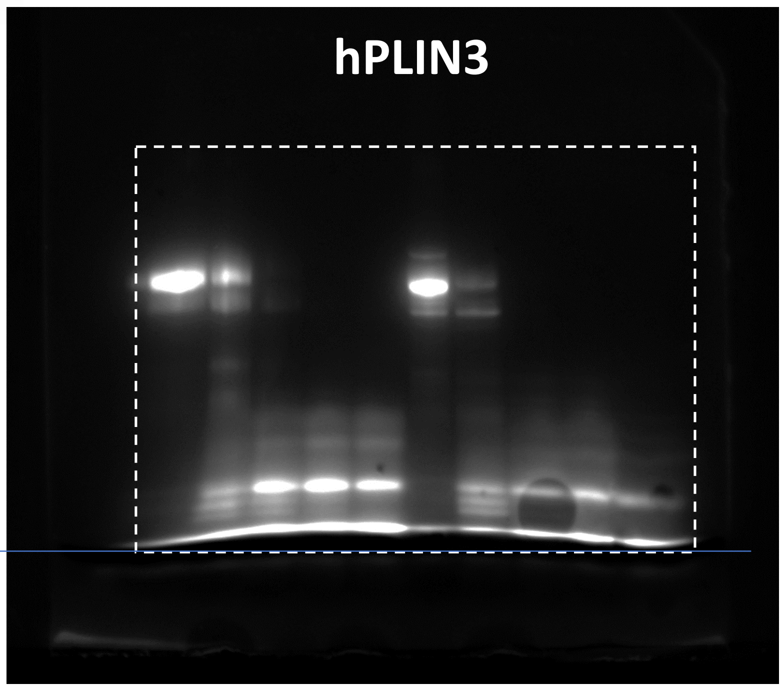

Supplement: SourceData FS3 — is the source file for Fig. S3. [file JCB_202403064_SourceDataFS3.pdf]
